# Long-Term Incubation of Lake Water Enables Genomic Sampling of Consortia Involving *Planctomycetes* and Candidate Phyla Radiation Bacteria

**DOI:** 10.1128/msystems.00223-22

**Published:** 2022-03-30

**Authors:** Alexander L. Jaffe, Maxime Fuster, Marie C. Schoelmerich, Lin-Xing Chen, Jonathan Colombet, Hermine Billard, Télesphore Sime-Ngando, Jillian F. Banfield

**Affiliations:** a Department of Plant and Microbial Biology, University of California, Berkeley, California, USA; b Laboratoire Microorganismes: Génome et Environnement (LMGE), UMR CNRS 6023, Université Clermont-Auvergne, Clermont-Ferrand, France; c Innovative Genomics Institute, University of California, Berkeley, California, USA; d Department of Earth and Planetary Science, University of California, Berkeley, California, USA; e Department of Environmental Science, Policy, and Management, University of California, Berkeley, California, USA; f Chan Zuckerberg Biohub, San Francisco, California, USA; University of California San Diego

**Keywords:** CPR bacteria, aster-like nanoparticles, lake microbiome, metagenomics, viruses

## Abstract

Microbial communities in lakes can profoundly impact biogeochemical processes through their individual activities and collective interactions. However, the complexity of these communities poses challenges, particularly for studying rare organisms such as Candidate Phyla Radiation bacteria (CPR) and enigmatic entities such as aster-like nanoparticles (ALNs). Here, a reactor was inoculated with water from Lake Fargette, France, and maintained under dark conditions at 4°C for 31 months and enriched for ALNs, diverse *Planctomycetes*, and CPR bacteria. We reconstructed draft genomes and predicted metabolic traits for 12 diverse *Planctomycetes* and 9 CPR bacteria, some of which are likely representatives of undescribed families or genera. One CPR genome representing the little-studied lineage “*Candidatus* Peribacter” was curated to completion (1.239 Mbp) and unexpectedly encodes the full gluconeogenesis pathway. Metatranscriptomic data indicate that some planctomycetes and CPR bacteria were active under the culture conditions, accounting for ∼30% and ∼1% of RNA reads mapping to the genome set, respectively. We also reconstructed genomes and obtained transmission electron microscope images for numerous viruses, including one with a >300-kbp genome and several predicted to infect *Planctomycetes*. Together, our analyses suggest that freshwater *Planctomycetes* are central players in a subsystem that includes ALNs, symbiotic CPR bacteria, and viruses.

**IMPORTANCE** Laboratory incubations of natural microbial communities can aid in the study of member organisms and their networks of interaction. This is particularly important for understudied lineages for which key elements of basic biology are still emerging. Using genomics and microscopy, we found that members of the bacterial lineage *Planctomycetes* may be central players in a subset of a freshwater lake microbiome that includes other bacteria, archaea, viruses, and mysterious entities, called aster-like nanoparticles (ALNs), whose origin is unknown. Our results help constrain the possible origins of ALNs and provide insight into possible interactions within a complex lake ecosystem.

## OBSERVATION

Freshwater lakes host diverse microbial communities that likely control ecosystem biogeochemistry (1, 2). Here, we established a laboratory culture based on an inoculum from Lake Fargette, France, a site chosen as part of a parallel study of enigmatic aster-like nanoparticles (ALNs) (3, 4). ALNs are enigmatic, organic, femtoplankton entities that exhibit bloom-like behavior in various freshwater and coastal environments (3, 4). In this experiment, microscopy showed that the proportion of ALNs increased substantially during incubation, from 26% to 36% of imaged objects (see Table S1 at https://zenodo.org/record/5362898#.YiuiRBPML5Y). To seek clues to the origins of ALNs and the organisms that they might associate with, and to better understand the lake ecosystem overall, we studied the culture using a combination of metagenomics, metatranscriptomics, and microscopy. In doing so, we recovered draft genomes for abundant and transcriptionally active *Planctomycetes* as well as CPR bacteria, phages, and eukaryotic viruses. Overall, we provide clues to associations and potential interactions among microbial groups in a lake ecosystem.

### Community composition and genome reconstruction.

**FIG 1:**
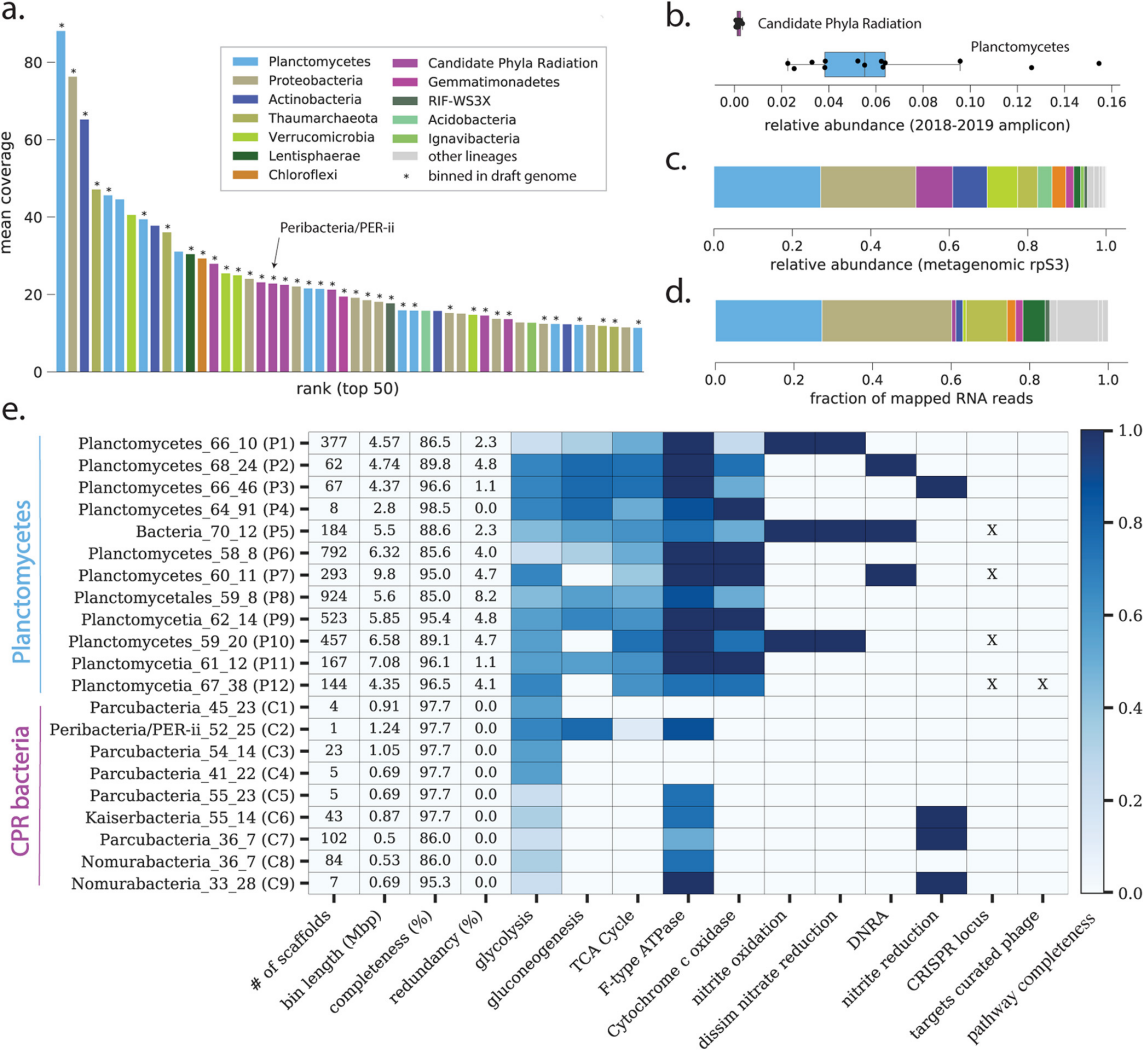
Long-term incubation enriched for members of the *Planctomycetes* and CPR bacteria. (a) Rank abundance curve based on ribosomal protein S3 (rps3) coverage for organisms recovered at the end of incubation. Asterisks indicate marker genes that were binned into genomes. (b) Relative abundance of CPR bacteria and *Planctomycetes* during monthly sampling of Lake Fargette based on 16S rRNA amplicon sequencing. Each point represents the relative abundance of CPR bacteria or *Planctomycetes* in a given month. (c) Overall community composition at the end of incubation, based on cumulative coverage of rpS3. Panel d displays the fraction of RNA reads from the end of incubation that could be mapped to genomes. (e) Sequence characteristics, metabolic predictions, and CRISPR-Cas loci information for genomes affiliated with the *Planctomycetes* and CPR bacteria. Cells with color fill indicate the fraction of key genes for each pathway (as defined by KEGGDecoder) that are present. X indicates genomes with CRISPR loci and if those loci contained spacers targeting at least one curated phage genome from the sample.

Concentrate from the 0.2-μm to 25-μm size fraction of the highly eutrophic Lake Fargette, France, was incubated at 4°C in the dark with filtered and sterilized lake water (<20 kDa). The incubation was performed under dark conditions to favor heterotrophic organisms associated with ALNs instead of phototrophic eukaryotes. After 31 months, DNA and RNA were extracted for metagenomic and transcriptomic analyses. DNA reads were assembled and the scaffolds profiled to identify ribosomal protein S3 (rpS3). Profiling using the predicted rpS3 protein sequences revealed that the enrichment was bacterially dominated, although several members of the *Thaumarchaeota* were present (Fig. 1a; see also Table S2 at https://zenodo.org/record/5362898#.YiuiRBPML5Y). The most abundant organisms overall were *Planctomycetes* (∼27% of overall rpS3 coverage), including the most abundant singular organism (Fig. 1 and Table S2). CPR bacteria were 5 of the top 25 most abundant organism groups (∼9% overall rpS3 coverage; Fig. 1 and Table S2). Compared to baseline abundances in Lake Fargette, these results indicated enrichment of these groups, particularly CPR bacteria, which were barely detectable in the lake (<1% relative abundance; Fig. 1b and Table S3).

Where possible, scaffolds were assigned to genome bins that ranged in quality from draft to nearly complete. The 48 genomes captured most of the phylogenetic diversity (76% of the most abundant rpS3 genes; Fig. 1a and Tables S2 and S4 at https://zenodo.org/record/5362898#.YiuiRBPML5Y); 12 genomes represent phylogenetically diverse *Planctomycetes*, including several from the *Planctomycetes* and *Phycisphaerae* classes (see Fig. S1 and Table S4). Metabolic reconstructions suggested that the *Planctomycetes* are primarily heterotrophs with the potential to oxidize nitrite or reduce nitrate in three cases (Fig. 1e). We also recovered 9 genomes of CPR bacteria, 8 of which were classified as “*Candidatus* Parcubacteria” (Fig. S1). The “*Candidatus* Parcubacteria” genomes encoded minimal metabolic capacities, consistent with symbiotic lifestyles (5). However, several had a phylogenetically distinct *nirK* gene that may play a role in denitrification or energy conservation (6) (Fig. 1e). Read mapping from a metatranscriptome collected contemporaneously with the metagenome suggested that some *Planctomycetes* and, to a lesser extent, CPR bacteria were actively transcribing under the culture conditions, with *Planctomycetes* and CPR accounting for about 27% and 1%, respectively, of RNA reads stringently mapping to the nonredundant set of genomes (Fig. 1d and Table S4). However, only a small proportion of total RNA reads (201,809 reads, or ∼0.2% of the total) mapped stringently to the genome set and passed filtering for non-mRNAs (see the supplemental material at https://zenodo.org/record/5362898#.YiuiRBPML5Y).

The ninth draft CPR genome was for a member of the undersampled CPR lineage “*Candidatus* Peribacteria.” We manually curated the original bin of 6 fragments into a single fragment that was circularized by a small, unbinned contig, and all scaffolding errors and local misassemblies were fixed (see Fig. S2 at https://zenodo.org/record/5362898#.YiuiRBPML5Y). The newly reported, fully curated 1,239,242-bp genome shares ∼89% similarity in its 16S rRNA gene and is largely syntenous with a closely related “*Candidatus* Peribacteria” genome from Rifle, Colorado (7), supporting the accuracy of both assemblies (see Fig. S3). Unlike the Rifle genomes, this peribacter likely cannot synthesize purines *de novo*. Other notable differences include the presence of vacuolar-type H^+^/Na^+^-transporting ATPase complex and the lack of genes for biosynthesis of mevalonate. Based on biosynthetic deficiencies, we conclude that this bacterium probably relies on other organisms for many building block but to a lesser extent than the “*Candidatus* Parcubacteria.” Supporting this idea, we observed that the peribacter genome encodes all gluconeogenesis enzymes, including fructose-1,6-bisphosphatase I, which was not found in the “*Candidatus* Parcubacteria” genomes (Fig. 1e).

**FIG 2:**
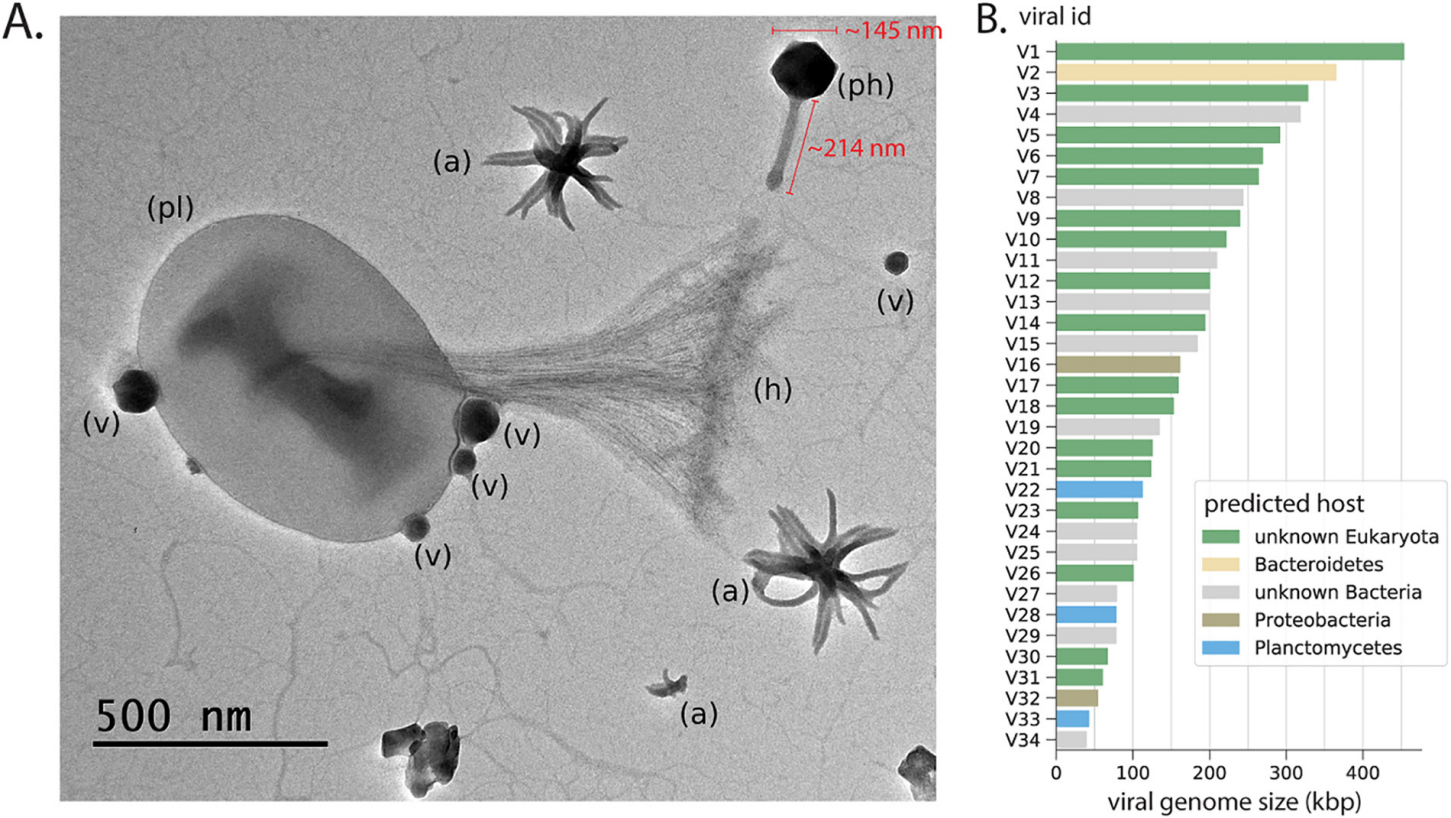
TEM imaging and viral genomics in the enrichment culture. (A) Featured in this image is a cell inferred to be a *Planctomycetes* (pl) with a characteristic stalk and holdfast (h). Attached to the cell are four phage particles (v) (two different sizes; thus, likely different phages). Also visible is one large tailed jumbo phage (ph) that is 145 nm in diameter with a 214-nm tail as well as several aster-like nanoparticles (ALNs) (a). (B) Genome sizes and predicted hosts for phages and eukaryotic viruses.

We reconstructed 12 phage sequences and 5 phage-like sequences, including 3 circularized genomes exceeding 100 kbp and 2 incomplete phage/phage-like fragments of >300 kbp (see Table S5 at https://zenodo.org/record/5362898#.YiuiRBPML5Y). Phylogenetic analyses of encoded terminase and capsid proteins suggested that the phages likely fall within the *Caudovirales*, which are known to include numerous tailed phages with large capsids (8). Additionally, we used phage gene content and analyses of bacterial CRISPR loci to infer that phages infect *Planctomycetes*, *Proteobacteria*, and *Bacteroidetes* (Fig. 2 and Table S5).

We also reconstructed large fragments of 17 eukaryotic viruses. Based on the phylogenetic placement of the major capsid protein and homologs of the poxvirus late transcription factor VLTF3, some viruses belonged to *Iridoviridae* (Betairidoviridae), extended *Mimiviridae*, *Phycodnaviridae*, and Pitho-like viruses (see Fig. S4 and S5 and Table S5 at https://zenodo.org/record/5362898#.YiuiRBPML5Y). Other viruses could only be classified at the superclade level, including those within a potentially novel *Phycodnaviridae*, *Asfarviridae*, *Megavirales* (PAM) clade, or did not contain either marker protein (see Fig. S4 and S5). Interestingly, we detected very little transcription of genes from phage or eukaryotic viruses (mean coverage, ≪1×), suggesting that they were not actively replicating in the incubated community (see Table S5). The lack of eukaryotes in the enrichment suggests that some of these particles have derived from the inoculum and persisted for over 2 years.

### Imaging of incubation community.

We imaged a diversity of cellular, cellular-like, and noncellular particles, as well as many ALNs, using transmission electron microscopy (TEM) (Fig. 2 and Fig. S6 at https://zenodo.org/record/5362898#.YiuiRBPML5Y). Based on the presence of the extracellular holdfast (9, 10), we infer that the cell imaged in Fig. 2 is likely a member of the *Planctomycetes*, with at least two attached distinct types of virus-like particles (VLPs). This finding is consistent with the high abundance of *Planctomycetes* in the enrichment as well as multiple phages predicted to infect them (∼18 to 100× coverage). We also imaged numerous tailed phages (see Fig. S6), including one with a 145-nm-diameter capsid and 214-nm tail (Fig. 2, ph). This large capsid size is consistent with those of jumbo phages with genomes in the 300-kbp range (8), two of which were reconstructed here (see Table S5). Despite these observations, TEM image counts suggested that the overall proportion of tailed phages decreased from 26% to less than 1% of imaged objects over the course of the incubation (see Table S1).

## DISCUSSION

*Planctomycetes* are globally distributed across freshwater ecosystems, where they are thought to play important roles in nitrogen and carbon cycling (11, 12). Our analyses expand genomic sampling for these organisms, and coenrichment suggests that they interact with CPR bacteria. Although we cannot establish a direct association between *Planctomycetes* and CPR bacteria from the current data, reported lifestyles for other CPR suggest that they are host cell-attached, at least at sometimes (13). Furthermore, the enrichment contains a similar level of diversity of both *Planctomycetes* and CPR, raising the possibility of species-specific associations (see Fig. S1 at https://zenodo.org/record/5362898#.YiuiRBPML5Y). As episymbionts, CPR would almost certainly influence the physiology of *Planctomycetes* cells, either via mutualistic interactions or parasitism, and, thus, the biogeochemical cycles that *Planctomycetes* mediate. The approach used in the current study could guide future coisolation of CPR bacteria of the “*Candidatus* Parcubacteria” and “*Candidatus* Peribacteria” lineages to test this hypothesis.

Like CPR bacteria, phages clearly impact their hosts and, thus, ecosystem structure and performance. It is intriguing that phages were maintained over the long incubation period, as they are often lost from laboratory cultures (14). A subset of the phages clearly infect *Planctomycetes*, the diversity of which may have enabled their sustained replication.

One motivation of our study was to seek clues to the origin of ALNs, and it is also intriguing to find them coenriched with *Planctomycetes* and CPR bacteria. Their defined morphologies suggest that they develop under genetic control; however, the genetic system responsible for producing ALNs remains unknown. We found no evidence for nucleic acid sequences from completely unknown organisms or mobile genetic elements or were not able to extract them with the methods used here. Thus, one possible interpretation is that ALNs originate from cooccurring bacteria and that *Planctomycetes* and CPR should be among candidates for further study.

Materials and methods can be found in the supplemental material at https://zenodo.org/record/5362898#.YiuiRBPML5Y.

### Data availability.

Read data and draft genomes from this study are available through NCBI at PRJNA757735. Genome accession information for the 48 bacterial and archaeal genomes is also listed in Table S3 at https://zenodo.org/record/5362898#.YiuiRBPML5Y. Custom codes for the described analyses are also available on GitHub (github.com/alexanderjaffe/aln-enrichment). All supplementary figures, tables, extended data files, and genomes (including phage/viral genomes) are also available through Zenodo (https://doi.org/10.5281/zenodo.5362897).
